# Effect of Different Mulches under Rainfall Concentration System on Corn Production in the Semi-arid Areas of the Loess Plateau

**DOI:** 10.1038/srep19019

**Published:** 2016-01-11

**Authors:** Xiaolong Ren, Peng Zhang, Xiaoli Chen, Jingjing Guo, Zhikuan Jia

**Affiliations:** 1 Institute of Water Saving Agriculture in Arid Areas of China, Northwest A&F University, Yangling, Shaanxi 712100, China; 2 Key Laboratory of Crop Physi-ecology and Tillage Science in Northwestern Loess Plateau, Ministry of Agriculture, Northwest A&F University, Yangling, Shaanxi 712100, China

## Abstract

The ridge and furrow farming system for rainfall concentration (RC) has gradually been popularized to improve the water availability for crops and to increase the water use efficiency (WUE), thereby stabilizing high yields. In the RC system, plastic-covered ridges are rainfall harvesting zones and furrows are planting zones. In this study, we optimized the mulching patterns for RC planting to mitigate the risks of drought during crop production in semi-arid agricultural areas. We conducted a four-year field study to determine the effects on corn production of mulching with 0.08-mm plastic film, maize straw, 8% biodegradable film, liquid film, bare furrow, and conventional flat (CF) farming. We found that RC significantly increased (*P* > 0.05) the soil moisture storage in the top 0–100 cm layer and the topsoil temperature (0–10 cm) during the corn-growing season. Combining RC with mulching further improved the rain-harvesting, moisture-retaining, and yield-increasing effects in furrows. Compared with CF, the four-year average yield increased by 1497.1 kg ha^–1^ to 2937.3 kg ha^–1^ using RC with mulch treatments and the WUE increased by 2.3 kg ha^–1^ mm^–1^ to 5.1 kg ha^–1^ mm^–1^.

The semi-arid areas of the Loess Plateau are of crucial importance for arid agriculture in China. In these areas, rainfall serves as the sole or main water source, and agricultural production depends mainly on natural precipitation. However, precipitation is relatively scarce in these areas, with an average annual amount of only about 450 mm, high variation among years, and uneven spatial and temporal distributions. The vast majority of the annual precipitation occurs between July and September, which does not match with the water demands of crops during their growth periods. In addition, the annual evaporation in these areas is more than 1500 mm, which frequently leads to severe droughts that affect crops, as well as causing large-scale water losses and soil erosion. Therefore, the sustainable development of agricultural production in the semi-arid farming areas of the Loess Plateau in China is still highly dependent on water-related factors. Improving the soil water content via field cultivation measures is a fundamental approach for addressing water constraints. According to the field water balance principle, enhancing water storage in the field soil reservoirs of arid areas demands surface runoff reduction, increased precipitation infiltration, and the inhibition of evaporation from the soil. Thus, increasing agricultural productivity in this region requires the optimized utilization of limited precipitation resources by implementing measures such as rain-harvesting and water storage, thereby improving the crop water use efficiency (WUE)^1^,^2^.

Based on the idea of *in situ* rain harvesting and utilization, ridge and furrow water-harvesting planting (also called root zone water-harvesting planting) is a type of micro-rain-harvesting system for rainfall concentration (RC), which was developed on the Loess Plateau and in the northwest areas of China where dryland farming is particularly common^3^. Ridges and furrows are alternated in the RC system, where the ridges are mulched with rain-harvesting materials and the furrows are planted with crops. The relationships and interactions among “furrows” and “ridges” comprise a system that regulates the water environment to meet the needs for crop growth and that exploits the full production potential of precipitation^4^. Many studies have shown that this method can harvest the surface runoff from precipitation^5^,^6^,^7^ as well as reducing evaporation^8^,^9^, increasing the daily radiation and soil temperature during the seedling stages of crops^10^, and thus improving the productivity of crops such as maize, potato, and winter wheat by effectively reducing water losses and enhancing the field WUE^11^,^12^. The yield-increasing effects of this system are strongly related to rainfall during the crop growth period^13^,^14^,^15^. A previous study demonstrated that this approach greatly improved the growth of trees in an Indian desert with annual rainfall of only 300 mm^16^. Combining planting and mulching methods with this approach can improve the crop yield and field WUE, as well as reducing the costs of production^17^. Therefore, studies of water-harvesting planting in agricultural fields can help to enhance the efficiency of precipitation use in dryland farming by improving the water-harvesting and planting methods employed to make full use of natural precipitation.

Corn (*Zea mays* L.) is one of the most popular grain crops in the semiarid Loess Plateau region of northwestern China. However, the corn productivity and yield are relatively low in these areas due to water shortages. In the present study, we conducted experiments at the Northwest Agriculture and Forestry University field test station in Pengyang, Ningxia Hui Autonomous Area (a typical semi-arid dry-farming area on the Loess Plateau), where we mulched the planting furrows in the RC planting system with maize straw, plastic film, biodegradable film, or liquid film to reduce soil water evaporation and to improve the rain-harvesting effects. In this four-year (2007 to 2010) field study, we analyzed the effects of RC planting on the field water temperature, yield, and WUE under different mulching patterns. Our results can help to optimize the selection of water-harvesting planting in fields, as well as providing a theoretical basis for improving water-harvesting planting to address practical issues related to the popularization of this method, especially the use of different mulching patterns in semi-arid farming areas that are severely affected by drought and water shortages.

## Results

### Soil moisture storage

**Figure 1:**
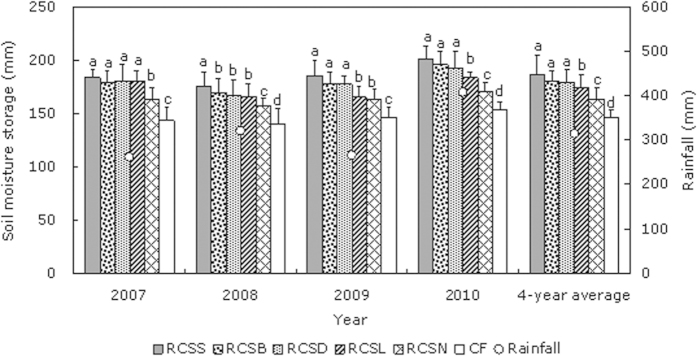
Effects of plastic-covered ridge and furrow rainfall concentration (RC) planting combined with mulching on the average soil moisture storage in the 0–100 cm depth from 2007 to 2010 during the corn growth period. Note: RC_SS_: 0.08 mm thick plastic film; RC_SD_: corn straw; RC_SB_: 8% biodegradable film; RC_SL_: liquid film; RC_SN_: bare furrow; CF: conventional flat (control). Different lowercase letters indicate significant differences among treatments. Error bars represent standard deviations.

In four consecutive years, the soil moisture storage was determined regularly in the 0–200 cm soil layer under different treatments during the whole growth period of spring corn. The results showed that in the semi-arid area of the Loess Plateau, the rainfall-harvesting effects of the ridges and furrows significantly improved the soil moisture storage (*P *< 0.05) in the 0–200 cm soil layer under all of the RC treatments compared with conventional flat (CF) farming method. After comparing the moisture storage levels in the different soil layers at depths of 0–200 cm, we found that the water increases occurred mainly at depths of 0–100 cm, whereas the spatial and temporal changes in moisture storage at depths below 100 cm were relatively small, and there were no significant (*P *< 0.05) differences between the RC treatments and CF, thus we not show the data of the 100–200 cm depth (Fig. 1). These results agreed with our previous study, where we applied movable rain shelters under different rainfall levels during the crop growth period^18^. During the overall spring corn growth period, the soil moisture storage levels in the furrows were significantly higher in the 0–100 cm layer (*P *< 0.05) when we used 0.08-mm plastic film (RC_SS_), 8% biodegradable film (RC_SB_), and maize straw (RC_SD_) mulches compared with the bare furrow (RC_SN_) treatment. Excluding 2009, the soil moisture storage levels in the furrows were significantly higher at the 0–100 cm depth (*P *< 0.05) when we used liquid film (RC_SL_) compared with the RC_SN_ treatment (Fig. 1). This may be explained by the spatial and temporal distributions of precipitation during 2009. In August 2009, the precipitation was 123.4 mm but the total precipitation during the corn growth period in 2009 was only 261.5 mm. During the growth corn period, major delays in precipitation undermined the capacity of the furrow mulching materials to retain soil water during the early growth period. In addition, the liquid films became damaged in the later growth period, which increased evaporation from the furrows. Therefore, throughout the whole corn growth period, there was no great benefit of using RC_SL_ in terms of water-harvesting or moisture retention compared with RC_SN_.

The RC planting furrows were mulched with different materials. During 2007–2010, we found that RC_SS_, RC_SB_, and RC_SD_ had the best water-harvesting and moisture-retaining effects, whereas RC_SL_ did not perform as well (Fig. 1). During the corn growth periods in 2007–2010, the annual mean soil moisture storage levels with RC_SS_, RC_SB_, RC_SD_, and RC_SL_ increased by 23.3 mm, 17.8 mm, 16.3 mm, and 10.6 mm, respectively, compared with the RC_SN_ treatment. Thus, compared with furrows that lacked mulching, RC planting with plastic films, crop straw, biodegradable film, and liquid film mulches greatly improved the rain-harvesting and moisture-retaining effects, probably because the mulching materials reduced the evaporation of soil water and improved the retention of moisture. Therefore, mulching the furrows in RC planting provided favorable conditions for enhancing the soil water conditions to meet the needs of crops.

### Soil temperature

**Figure 2:**
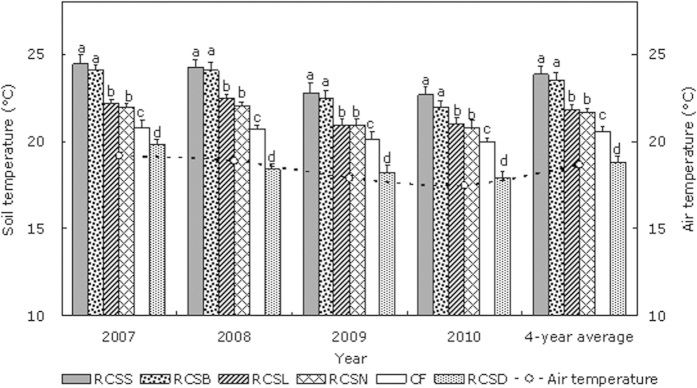
Effects of plastic-covered ridge and furrow rainfall concentration (RC) planting combined with mulching on the mean daily soil surface temperature in the 0–10 cm depth from 2007 to 2010 during the corn growth period. Note: RC_SS_: 0.08 mm thick plastic film; RC_SD_: corn straw; RC_SB_: 8% biodegradable film; RC_SL_: liquid film; RC_SN_: bare furrow; CF: conventional flat (control). Different lowercase letters indicate significant differences among treatments. Error bars represent standard deviations.

We found that the soil temperature in the furrows was affected significantly by mulching to depths of 5 cm (*P *< 0.05) (Fig. 2). During 2007–2010, the differences among treatments had the same trend in specific years. RC_SS_ and RC_SB_ had the best warming effects whereas RC_SL_ and RC_SN_ did not perform as well. Compared with CF, RC_SD_ actually had a significant cooling effect. At 30 days after sowing, the average soil temperatures at a depth of 5 cm were all significantly higher using RC_SS_, RC_SB_, RC_SL_, and RC_SN_ (*P *< 0.05) compared with CF. During 2007–2010, the average annual temperature increased by 3.3 °C, 3.0 °C, 1.3 °C, and 1.0 °C under RC_SS_, RC_SB_, RC_SL_, and RC_SN_ compared with CF, respectively, whereas the temperature was 1.8 °C lower when using RC_SD_ compared with CF, which was possibly because the structure of the ridges and furrows increased radiation in the field. Moreover, RC_SS_ and RC_SB_ had the greatest effects on heat retention, with the most remarkable warming effects. The liquid film formed a thin black layer on the furrows, which made the diurnal temperature increase relatively rapidly as the environmental temperature rose but it also decreased relatively rapidly as the temperature declined. Therefore, over 30 days, the average diurnal temperature increased slightly under RC_SL_ compared with RC_SN_, but the difference was not significant (*P *< 0.05) (Fig. 2). In addition, RC_SD_ had significant cooling effects compared with CK, which was because the straw mulch had almost no photo permeability and a relatively poor thermal conductivity layer formed at the soil surface, thereby inhibiting heat exchange between the soil and air.

These results indicate that the topsoil temperature was effectively improved by RC planting and the warming effects were enhanced further by mulching the furrows with plastic film and biodegradable film, which provided highly favorable conditions for seedling growth in the experimental area where the average temperature was only 6.1 °C.

### Grain yield and WUE

**Figure 3:**
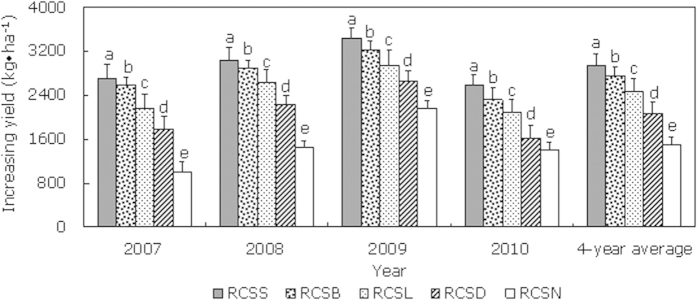
Yield increases obtained under plastic-covered ridge and furrow rainfall concentration (RC) planting combined with mulching from 2007 to 2010 during the corn growth period compared with the conventional flat (CF) treatment. Note: RC_SS_: 0.08 mm thick plastic film; RC_SD_: corn straw; RC_SB_: 8% biodegradable film; RC_SL_: liquid film; RC_SN_: bare furrow; CF: conventional flat (control). Different lowercase letters indicate significant differences among treatments. Error bars represent standard deviations.

The results of field experiments in four consecutive years showed that RC planting significantly (*P *< 0.05) enhanced the spring corn grain yield. During 2007–2010, all of the treatments increased the yield (Table 1). Compared with CF, RC_SS_ and RC_SB_ had the best effects on the yield, followed by RC_SL_ and RC_SD_. Compared with the other mulching treatments, RC_SN_ had a relatively small effect on increasing yield, but compared with CF, the yield still improved by a relatively large amount (Fig. 3). The four-year average grain yields under RC_SS_, RC_SB_, RC_SL_, RC_SD_, and RC_SN_ increased by 2937.3 kg ha^–1^, 2745.1 k ha^–1^, 2455.9 kg ha^–1^, 2066.9 kg ha^–1^, and 1497.1 kg ha^–1^, respectively, i.e., 63.9%, 59.7%, 53.4%, 45.0%, and 32.6% increases compared with CF. Thus, the yield increases were highly significant. Compared with no mulching in the furrows (RC_SN_), the average annual yields under RC_SS_, RC_SB_, RC_SL_, and RC_SD_ increased by 23.6%, 20.5%, 15.8%, and 9.3%, respectively. Compared with RC_SN_, the yield increase with RC_SD_ was relatively low, probably because mulching the furrows with straw reduced the soil surface temperature and this affected the economic yield. The RC treatments significantly (*P *< 0.05) improved the WUE for spring corn (Table 1), where the four-year average WUE values for RC_SS_, RC_SB_, RC_SL_, RC_SD_, and RC_SN_ improved by 5.1 kg ha^–1^ mm^–1^, 4.7 kg ha^–1^ mm^–1^, 4.2 kg ha^–1^ mm^–1^, 2.4 kg ha^–1^ mm^–1^, and 2.3 kg ha^–1^ mm^–1^, respectively, compared with CF, i.e., increases of 38.0%, 35.1%, 31.3%, 17.9%, and 17.2%. This indicated that the soil moisture storage increased with RC planting, thereby improving the soil water environment needed for crop growth, which enhanced the economic yield of spring corn, reduced the area available for evaporation from fields, and decreased overall evaporation from the soil. Moreover, RC combined with different materials in furrows greatly improved the effects of rain-harvesting, evaporation inhibition, and moisture conservation, which further enhanced the field WUE. There was no significant difference in the WUE under RC_SD_ and RC_SN_, which was possibly because mulching the furrows with straw decreased the temperature of the soil and this influenced the effective utilization of soil water and yield formation by spring corn, and thus RC_SD_ had no obvious advantages in terms of the field WUE compared with RC_SN_.

## Discussion

### Soil moisture and temperature

Suitable soil moisture and temperature conditions are essential for obtaining high yields during crop production^19^,^20^. Previous studies indicate that planting patterns have great effects on the field water conditions during the early stage of the corn growth period^21^,^22^,^23^. Thus, favorable temperatures can significantly promote seed germination and dry matter accumulation in crops at 30 days after sowing^24^. Soil temperatures below 15 °C can detrimentally affect the growth of spring corn^25^ and temperatures below 8 °C can severely inhibit its growth^26^. Many studies suggest that the changes in soil moisture and temperature caused by planting systems are the main reasons for crop yield differences in rainfed areas^27^. The results of our study also indicate that the improvements in soil moisture and temperature with the RC system were beneficial for corn growth, thereby resulting in higher yields.

RC planting can effectively improve the soil moisture and thermal conditions during the early stage of corn growth, thereby enhancing the development of field cultivation in the semi-arid farming areas of the Loess Plateau^17^,^18^. The soil temperature is one of the main factors that can influence corn growth during the early stage, where the base temperature is 8 °C for corn, but growth is likely to be severely impaired when the temperature is less than 15 °C. In our study, both the daily mean soil temperature and maximum soil temperature at a depth of 5 cm were higher than 15 °C under all treatments in the VE to V1 stages, whereas the daily minimum temperature was lower than 15 °C under all treatments. This may indicate that improving the daily minimum temperature during the early growth period is important for enhancing growth. Song *et al.*^27^ studied the effects of different ridging times on the soil water temperature in the rainfed areas of northeast China, where they detected significant differences in the soil surface temperature under various treatments during the early growth period for corn. From the sowing and seed germination stage to the early jointing stage, the average minimum diurnal topsoil temperature at a depth of 5 cm increased by 1.2 °C and 1.1 °C under flat ridge and flat treatments, respectively, whereas the corresponding average maximum diurnal topsoil temperature decreased by 2.4 °C and 2.6 °C, respectively. This may be attributable to the relatively low spring temperatures in northeast China at high altitudes. Li *et al.*^28^ studied the effects of different sand-mulching patterns in rain-harvesting planting furrows on the field water and temperature conditions for spring corn based on field experiments in a semi-arid farming area. Their results suggested that sand mulching significantly improved water storage in the rain-harvesting planting furrows at soil layer depths of 0–140 cm and the average soil temperature increased by about 1 °C during the crop growth period compared with that under flat treatments. In the present study, in a semi-arid farming area of the Loess Plateau with an average annual precipitation of 450 mm and average annual evaporation of 1750 mm, we found that RC planting greatly enhanced the water storage at soil depths of 0–100 cm for spring corn, as well as increasing the soil surface temperatures during the early growth period of corn, which agreed with the results obtained in a previous research^17^. However, after mulching the planting furrows with different materials, the warming and water-harvesting effects were further intensified, especially when compared with the no-mulching treatment. Mulching with plastic film and biodegradable film had the greatest effects on the soil water content and temperature, probably because these mulching materials simultaneously reduced soil water evaporation and any heat losses due to soil water evaporation. The increased soil water content also enhanced the specific heat in the soil layers, which was favorable for soil heat retention.

### Yield and WUE

Differences in the corn yield occurred among years due to meteorological variation. However, from 2007 to 2010, RC planting significantly enhanced the corn yield compared with CF, where mulching the furrows with plastic film and biodegradable film obtained the best yield increases. The mulching treatments increased the WUE and the RC_SS_ treatment produced the highest WUE over the four years. Mulching with plastic film clearly reduced soil evaporation, decreased evapotranspiration and thus the water passing through the crop by transpiration increased^9^. Previous studies have shown that planting patterns can affect the soil water content and soil temperature, thereby modifying the crop yield and field WUE^29^,^30^. Song *et al.*^27^ studied the effects of field ridging at different times on the corn yield in a dryland farming area of northeast China at a high latitude, where the results of their three-year field experiments showed that under ridging treatments at the early jointing stage, the average corn yield increased by 302.2 kg ha^–1^ and 552.3 kg ha^–1^ compared with ridge treatments before sowing and conventional flat treatments, respectively, and thus field ridging improved the corn yield. Zhou *et al.*^31^,^32^ also showed that RC planting in fields greatly increased the soil temperature, reduced evaporation, and improved the grain yield for corn. We found that combining the RC system with mulching greatly increased corn production, especially the R_ss_ treatment, which was mainly because the plastic film reduced soil evaporation and increased topsoil temperature, as shown previously by Ren *et al.*^4^ The improved soil water content and temperature promoted the growth of crop roots, thereby enhancing the root distribution across the soil profile. Improvements in the soil water content and temperature also enhanced the ability of maize plants to extract more nutrients and water from the soil^15^. In addition, the greater soil moisture content and higher temperature might have led to increases in microbial activity, with a higher microbial biomass. According to Li *et al.*, the soil moisture conditions have important effects on the microbial biomass carbon^10^. Consequently, this planting pattern may be a key technique for substantially increasing maize productivity in areas with low air temperatures, limited water availability, and poor soil fertility. We found that plastic-covered RC planting greatly enhanced the spring corn yield and improved the field WUE, and the use of different mulching materials in the furrows improved the yield further.

In addition to these improvements in the corn yields, the cost-benefit effectiveness of the RC system must be considered. Based on the local farming system and the price of the mulching materials used in the four-year field experiments, the cost of the RC_SN_ (bare furrow) treatment would be about 585 RMB Yuan ha^–1^ (assuming that the conversion rate between US$ and RMB Yuan is about 1:6.3) higher than the CF treatment (including the costs of labor and plastic film) each year. The corresponding economic benefit would increase by about 2000–2300 RMB Yuan ha^–1^ (assuming a corn price of about 1.5 RMB Yuan kg^–1^) in an average year. The costs of the RC_SS_ (0.08 mm thick plastic film), RC_SD_ (corn straw), RC_SB_ (8% biodegradable film), and RC_SL_ (liquid film) treatments would be about 1000–1200, 1200–1300, 1000–1500, and 1300–1500 RMB Yuan ha^–1^ higher than the CF treatment, respectively. The corresponding increases in net economic benefit (excluding the costs of labor and mulching materials) would be about 3200–3400 for RC_SS_, 3000–3100 for RC_SB_, 2100–2300 for RC_SD_, and 2500–2600 for RC_SL_ compared with the CF treatment. Thus, although this system requires some investment, this can be offset by growing cash crops and this option has high potential for increasing crop sustainability in dryland farming systems. In the experimental area, farmers often give little consideration to the costs of labor in agricultural production, but the labor costs include the preparation of ridges and mulching the furrows. In addition, biodegradable film and straw are relatively low-cost and environmentally friendly materials. Therefore, we conclude that mulching furrows with crop straw and biodegradable film could be a suitable model for corn production by small-holding farmers in semiarid areas.

## Conclusions

In the semi-arid farming areas of the Loess Plateau in China, the effects of rain-harvesting planting on yield increases can be improved further by mulching the planting furrows with plastic film, biodegradable film, straw, and liquid film. We recommend that mulching the furrows with crop straw and biodegradable film should be adopted in the semi-arid farming areas of the Loess Plateau based on considerations of white pollution control and reducing film residues in the soil.

## Materials and Methods

### Study site description

**Figure 4:**
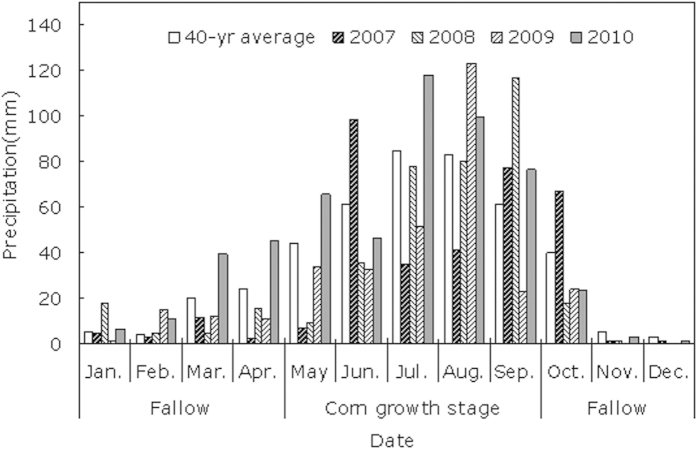
Yearly average distribution of precipitation during the corn growth period from 2007 to 2010 at Ganjing experimental station of Northwest A&F University.

The field experiments were conducted over four growing seasons from April 2007 to October 2010 at the Dryland Agricultural Research Station of Northwest A&F University, which is located in Baiyang town (35°79′N, 106°45′E, and 1800 m asl), Pengyang County, Ningxia Hui autonomous region, China. The station is located in the hilly area of the Loess Plateau, which is characterized by a temperate semi-arid continental monsoon climate at an altitude of 1800 m. The annual mean temperature was 6.1 °C. The annual mean pan evaporation was 1750 mm and the annual mean precipitation was 435.0 mm, 60% of which occurred in July, August, and September. The total yearly sunshine duration was 2518.2 h and the frost-free period was 155 days. The distribution of rainfall during the experimental tests is shown in Fig. 4. According to the average annual rainfall during the last four decades from 1970 to 2010, we treated 2007, 2008, and 2009 as years of water deficit, when the annual rainfall rates were 82.3 mm, 52.6 mm, and 106.8 mm less than the average annual rainfall (435.0 mm), respectively. By contrast, 2010 was a year of water abundance when the annual rainfall was 102.0 mm greater than the average annual rainfall. In 2007, 2008, 2009, and 2010, the total rainfall rates during the corn growth period (from late April to late September) were 260.5 mm, 320.0 mm, 265.1 mm, and 407.3 mm, respectively, whereas during the winter fallow period (from early October to mid-April in the following year), the total rainfall rates were 92.2 mm, 62.4 mm, 63.1 mm, and 129.7 mm, respectively. The field experiment was conducted on flat ground with loessal soil, which had relatively low fertility. In April 2007, the soil layers had the following physical and chemical properties at the beginning of the experiment: soil volume weight = 1.39 g cm^–3^, organic matter content = 8.41 g kg^–1^, total nitrogen concentration = 0.52 g kg^–1^, alkali-hydrolyzable nitrogen concentration = 51.62 mg kg^–1^, readily available phosphorus concentration = 9.21 mg kg^–1^, readily available potassium concentration = 114.27 mg kg^–1^, and pH value = 8.21. The preceding crop was spring corn (sown in 2006) and the testing fields were rendered fallow from October 2006 until April 2007.

### Experimental design and treatments

**Figure 5:**
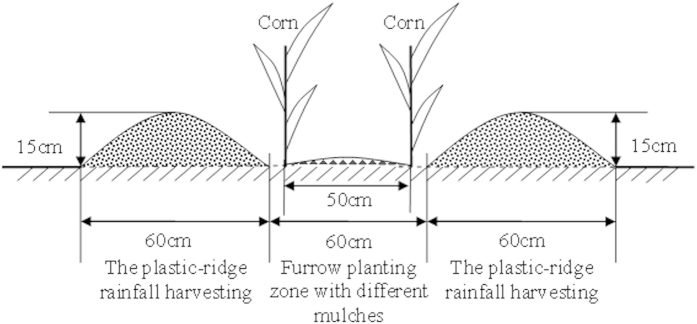
Schematic diagram showing the plastic-covered ridge and furrow rainfall concentration method combined with mulching.

The field experiment employed ridge and furrow RC planting. The ridges (width = 60 cm, side-slope = 40°) served as the rainfall harvesting zone and the furrows as planting zones. The experiment employed a randomized complete block design. There were six treatments and each had three replicates (Table 2). Excluding the controls, in all of the treatments, the ridges were covered with 0.08 mm thick plastic film and the furrows were mulched as follows: 0.08 mm plastic film for RC_SS_, maize straw for RC_SD_, 8% biodegradable film for RC_SB_, liquid film for RC_SL_, bare furrow for RC_SN_, and CF. In the field tests, the area of each planting catchment was 4 × 10 m where the ridge: furrow width ratio was 1:1, the ridge and furrow widths were 60 cm, the runoff-generating (ridge) area was 4 × 0.6 m (Fig. 5), and the ridge height was 15 cm. The internal distance between a ridge and corn furrow was 5 cm, and the distance between individual plants in CF and all of the RC treatments was 30 cm with a row distance of 60 cm. The side rows of each catchment served as protective rows. Plastic films that functioned as water isolation belts were buried at a depth of 2 m along the edges of each catchment, thereby preventing water infiltration among catchments.

Spring corn was sown on April 30 in 2007 and 2008, May 1 in 2009, and April 28 in 2010, and then harvested on September 30 in 2007 and 2008, October 1 in 2009 and September 29 in 2010. The plant population was thinned to a density of 55555.6 plants ha^–1^. Prior to planting, the ridges were banked up with soil and covered with plastic film (polyethylene film: width = 1.0 m, thickness = 0.08 mm), and the planting belts or furrows were then leveled. Farmyard manure was applied at a rate of 37,500 kg ha^–1^, N fertilizer at a rate of 150 kg ha^–1^, and P_2_O_5_ fertilizer at a rate of 75 kg ha^–1^. The manure was spread evenly over the furrows and then plowed into the soil layer. In each ridge and furrow plot, the sowing and fertilizer application rates were the same as those in the control plots based on the land area. The sowing and nitrogen application rates in each row under RC planting were twice those for the control, but they were actually the same based on the total plot area, including the ridges and furrows. Cultivar “Yuyu22” was used in the experiments, which is suitable for spring sowing in this area. Two seeds were sown per hole at a sowing depth of about 5 cm. In approximately 98% of cases, the root depth reached 100 cm. Thinning was conducted after seed germination, where one seedling per hole was retained at the trefoil stage. During the large bell stage, a urea (with a nitrogen content of 46%) topdressing was applied at 300 kg ha^–1^ by single hole topdressing with an average amount of 5.4 g per plant, but no further topdressing or irrigation was applied. During the overall maize growth period, weed and pest control were performed as necessary and soil scarification was conducted after every rainfall event to prevent soil hardening in the planting area. The plastic film used in this study was manufactured by Shanxi Yuncheng Plastic Factory. The biodegradable film was manufactured by Shaanxi Huayu High-tech and Biological Limited Company. The maize straw was locally produced dry straw, which was used to evenly mulch the furrows at 9000 kg ha^–1^. Liquid film produced by Zhejiang Aiketai Investment Limited Company was diluted with water at a ratio of 1:9, as recommended by the manufacturer, and sprayed evenly into the planting furrows using a sprayer.

### Measurements and data analysis

The soil moisture storage was determined at soil depths of 0–200 cm. Sampling was conducted with one sample per 10 cm for 0–20 cm and one sample per 20 cm at subsequent depths. Each sample was replicated three times. In the RC planting belts, sampling was conducted in planting furrows at half the furrow width, on the boundaries of ridges and furrows, and half the furrow width under the rain-harvesting ridges. For CF, samples were collected halfway between the planting rows. Soil samples were obtained by auger boring (with a diameter of 0.08 m). The soil samples were then dried before further analyses. Samples were collected during spring corn sowing and at 30, 60, 90, and 120 days after sowing. The final water determination during the overall corn growth period was made when the corn was harvested.

The requisite indices were calculated according to the following formulae.


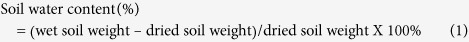


The soil water storage (in mm) measured vertically at a specific depth was calculated using Eq. (2), as follows:


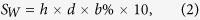


where *S*_*W*_ (mm) is the average soil water storage value, *h* (cm) is the soil layer depth, *d* (g cm^–3^) is the soil bulk density in a specific soil layer, and *b*% is the percentage gravimetric soil water content.

WUE was calculated according to the field water balance principle, as follows 28,33,34.

In RC planting areas:


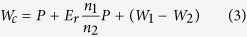



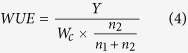


In conventional flat planting areas:


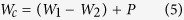



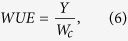


where *W*_*c*_ (mm) is the crop water consumption, *WUE* (kg ha^–1^ mm^–1^) is the water use efficiency in fields, *W*_*1*_ and *W*_*2*_(mm) are the water storage rates determined at a soil depth of 200 cm in two consecutive samplings (moisture storage under ridge and furrow planting was calculated as the average water storage in ridges and furrows), *E*_*r*_ is the ratio of the runoff yield at the ridge surface relative to the rainfall, i.e., the rain-harvesting efficiency or runoff efficiency (the average runoff efficiency for the film-mulched ridges was 0.87^10^), *P* (mm) is the total precipitation during the overall maize growth period, *n*_*1*_ and *n*_*2*_(cm) are the ridge and furrow widths, respectively, and *Y* (kg ha^–1^) is the seed yield obtained from the total area of ridges and furrows. The WUE of the grain yield can be calculated according to Eq. (6).

Geothermometers were placed in the rain-harvesting furrows and the middle of the corn-sowing rows in conventional flat planting areas at a soil layer depth of 5 cm to determine the soil temperature. After sowing, temperature data were recorded daily for 30 consecutive days. Each daily temperature observation was obtained from 8:00 am to 8:00 pm and data were recorded once every 2 h. The air temperature was recorded in the surrounding field at the same time as the soil temperature was recorded.

After maturity, rows of representative, evenly growing corn plants were selected to determine the yield components (including the spike number per unit area, grain number per spike, and hundred-seed weight) and the economic yield of corn was calculated according to these components.

The experimental data were analyzed with SAS and Excel. F-tests were conducted and multiple comparisons were performed using least significant difference tests (*P* ≤ 0.05).

## Additional Information

**How to cite this article**: Ren, X. *et al.* Effect of Different Mulches under Rainfall Concentration System on Corn Production in the Semi-arid Areas of the Loess Plateau. *Sci. Rep.* **6**, 19019; doi: 10.1038/srep19019 (2016).

## Figures and Tables

**Table 1:** Effects of ridge and furrow rainfall concentration (RC) combined with mulching on the corn grain yield, yield components, and water use efficiency (WUE) during 2007–2010.

Year	Treatment	Yield (kg ha^–1^)	100-seed weight (g)	Seed number per ear	*ET*(mm)	*WUE*(kg ha^–1^ mm^–1^)
2007	RC_SS_	6857.0 ± 299.6a	31.1 ± 0.7a	335.6 ± 12.1a	374.7	18.3 ± 0.8a
	RC_SD_	5940.2 ± 221.4b	30.4 ± 0.5a	292.0 ± 10.3b	373.6	15.9 ± 0.6c
	RC_SB_	6737.3 ± 169.3a	29.5 ± 0.6a	345.4 ± 14.7a	378.5	17.8 ± 0.4ab
	RC_SL_	6316.4 ± 195.4b	28.4 ± 1.0ab	336.4 ± 11.7a	369.4	17.1 ± 0.5b
	RC_SN_	5163.0 ± 234.5c	25.2 ± 0.7b	305.2 ± 9.6b	333.1	15.5 ± 0.7c
	CF	4153.4 ± 182.4d	24.1 ± 0.9b	257.4 ± 11.5c	314.7	13.2 ± 0.6d
2008	RC_SS_	7916.7 ± 304.1a	32.9 ± 1.1a	369.8 ± 11.8a	399.8	19.5 ± 0.8a
	RC_SD_	7104.2 ± 256.3b	31.4 ± 1.0a	330.2 ± 8.6c	413.0	16.2 ± 0.6b
	RC_SB_	7757.0 ± 208.4a	31.5 ± 0.8a	368.2 ± 10.5a	410.4	18.9 ± 0.5a
	RC_SL_	7514.0 ± 240.2ab	29.7 ± 0.9a	375.3 ± 14.3a	410.6	18.3 ± 0.6ab
	RC_SN_	6319.5 ± 224.3c	26.5 ± 0.9b	356.5 ± 11.8a	392.5	16.1 ± 0.6b
	CF	4881.8 ± 208.4d	23.4 ± 1.1c	318.8 ± 10.6d	341.4	14.3 ± 0.6c
2009	RC_SS_	7638.5 ± 238.6a	31.9 ± 0.9a	358.0 ± 12.8a	406.3	17.8 ± 0.6a
	RC_SD_	6855.2 ± 198.8b	29.5 ± 1.2a	346.4 ± 11.4a	418.0	15.4 ± 0.5b
	RC_SB_	7419.3 ± 212.1a	31.2 ± 1.0a	358.5 ± 12.3a	398.9	17.6 ± 0.5a
	RC_SL_	7150.6 ± 172.3ab	28.4 ± 1.3a	375.5 ± 10.8a	390.7	17.3 ± 0.4a
	RC_SN_	6357.2 ± 185.6c	26.7 ± 0.8b	358.0 ± 12.0a	392.4	15.2 ± 0.5b
	CF	4211.1 ± 159.1d	22.7 ± 0.7c	277.1 ± 11.6b	307.4	12.7 ± 0.5c
2010	RC_SS_	7725.6 ± 227.4a	32.9 ± 0.9a	351.1 ± 12.7a	419.9	18.4 ± 0.5a
	RC_SD_	6756.7 ± 212.6b	31.4 ± 1.2a	320.6 ± 10.4b	430.4	15.7 ± 0.6b
	RC_SB_	7455.6 ± 196.3a	32.5 ± 0.8a	346.9 ± 11.6a	409.6	18.2 ± 0.5a
	RC_SL_	7231.5 ± 153.4ab	30.6 ± 0.7a	356.3 ± 10.5a	406.3	17.8 ± 0.6a
	RC_SN_	6537.4 ± 165.7c	27.5 ± 1.3b	357.4 ± 9.3a	408.6	16.0 ± 0.4b
	CF	5142.3 ± 162.3d	25.4 ± 1.1c	300.5 ± 11.5c	380.9	13.5 ± 0.6c
Mean	RC_SS_	7534.5 ± 267.4a	32.2 ± 0.9a	353.6 ± 12.4a	400.2	18.5 ± 0.7a
	RC_SD_	6664.1 ± 222.3b	30.7 ± 1.0a	322.3 ± 10.2b	408.8	15.8 ± 0.6b
	RC_SB_	7342.3 ± 196.5a	31.2 ± 0.8a	354.8 ± 12.3a	399.4	18.1 ± 0.5a
	RC_SL_	7053.1 ± 190.3ab	29.3 ± 1.0a	360.9 ± 11.8a	394.3	17.6 ± 0.5a
	RC_SN_	6094.3 ± 202.5c	26.5 ± 0.9a	344.3 ± 10.7a	381.7	15.7 ± 0.6b
	CF	4597.2 ± 178.1d	23.9 ± 1.0a	288.5 ± 11.3c	336.1	13.4 ± 0.6c

Note: RC_SS_: 0.08 mm thick plastic film; RC_SD_: corn straw; RC_SB_: 8% biodegradable film; RC_SL_: liquid film; RC_SN_: bare furrow; CF: conventional flat (control); ET: evapotranspiration. Means ± SE within a column followed by the same letters do not differ significantly at the 5% level (Duncan’s multiple range test).

**Table 2:** Field experiment treatments.

Treatment	Ridges with mulching	Furrows with mulching	Planting pattern
RC_SS_	0.08 mm plastic film	0.08 mm plastic film	Rainfall concentration
RC_SD_	0.08 mm plastic film	Corn straw (9000 kg ha^–1^)	Rainfall concentration
RC_SB_	0.08 mm plastic film	8% biodegradable film	Rainfall concentration
RC_SL_	0.08 mm plastic film	Liquid film	Rainfall concentration
RC_SN_	0.08 mm plastic film	Bare furrow	Rainfall concentration
CF(control)	Bare flat soil	Bare flat soil	Conventional flat
